# The Making of the Women in Biology Forum (WiB) at Bioclues

**DOI:** 10.1371/journal.pcbi.1003436

**Published:** 2014-01-16

**Authors:** Reeta Rani Singhania, Dhatri Madduru, Pranathi Pappu, Sameera Panchangam, Renuka Suravajhala, Mohanalatha Chandrasekharan

**Affiliations:** Bioclues Organization, IKP Knowledge Park, Picket, Secunderabad, India

## Abstract

The Women in Biology forum (WiB) of Bioclues (India) began in 2009 to promote and support women pursuing careers in bioinformatics and computational biology. WiB was formed in order to help women scientists deprived of basic research, boost the prominence of women scientists particularly from developing countries, and bridge the gender gap to innovation. WiB has also served as a platform to highlight the work of established female scientists in these fields. Several award-winning women researchers have shared their experiences and provided valuable suggestions to WiB. Headed by Mohanalatha Chandrasekharan and supported by Dr. Reeta Rani Singhania and Renuka Suravajhala, WiB has seen major progress in the last couple of years particularly in the two avenues Mentoring and Research, off the four avenues in Bioclues: *Mentoring, Outreach, Research and Entrepreneurship (MORE).*

In line with the Bioclues vision for bioinformatics in India, the WiB Journal Club (JoC) recognizes women scientists working on functional genomics and bioinformatics, and provides scientific mentorship and support for project design and hypothesis formulation. As a part of Bioclues, WiB members practice the group's open-desk policy and its belief that all members are free to express their own thoughts and opinions. The WiB forum appreciates suggestions and welcomes scientists from around the world to be a part of their mission to encourage women to pursue computational biology and bioinformatics.

## Introduction

Bioclues is a nonprofit organization that was founded in 2005 by Drs. Prashanth Suravajhala and Pritish Varadwaj. It is the only official affiliate of the International Society for Computational Biology (ISCB) and the Asia Pacific Bioinformatics Network (APBioNet) in India. Bioclues is proud to have over 3,500 members in 40 countries. It has provided mentorship to over 400 students and collaborates with ∼12 universities and organizations in eight countries. Bioclues began with a vision of training at least 2,000 biologists in bioinformatics by the year 2020, but it was not until 2009 when the board realized that the inclusion of women trainees was essential to meeting this goal that the WiB forum was formed as a subgroup of Bioclues.

## Objectives

The WiB Journal Club (JoC) is a group of 30 women members who are students, young researchers, and established scientists who share a common interest in bioinformatics research. Through monthly meetings members discuss research articles, project designs, and opportunities to pursue new research areas. Further, they encourage women biologists to take up entrepreneurship per their interests. Leading women researchers participate in these discussions and provide unique insights and advice from their own experiences.

## Achievements and Accolades

The year 2012 has seen major breakthroughs for women scientists in general and WiB members in particular. The Organization for Women in Science for the Developing World (OWSD) recognized the work of Dr. Priya Mahadevan from S. N. Bose National Centre for Basic Sciences, India and Dr. Ilkay E. Orhan of the Medical and Health Sciences Department at Gazi University, Ankara, Turkey. These award-winning scientists shared their experiences with the WiB forum.

WiB invites women to discuss their research proposals and offers them a platform to work on it through Bioclues virtual projects. The most recent achievement, in October 2013, was the publication of an article titled “Women Biologists in India: Challenges” in *Current Science*. In May 2013, WiB published “Studies on 8-tertbutyl Caffeine: An In Silico Approach to Mechanistic Studies” in the *International Journal of Computational Biology* and in February 2013 “Toward Understanding the Role of p53 in Cardiovascular Diseases” with Peruvian collaborator Dr. Silvia Vasquez from El Instituto Peruano de Energía Nuclear (IPEN) in the *Journal of Biomedical Science and Engineering*. Another WiB member published her research on “Modeling Caffeine and Xanthine Analog Intermediates for Cancer Cell Line Studies” in May 2012.

At present, WiB members are contributing to several ongoing projects and a few more of its articles are in press, including “Next Generation Sequencing Analysis of Lung Cancer Datasets: A Functional Genomics Perspective,” “Protein Interactors of the Cellular Tumor Antigen p53 with Response to Ionizing Radiation,” and “In Silico Analysis of Laccases.” There is also a WiB team working on “Prediction of Proteins Implicated in Xanthinuria.”

The WiB members have been a part of the team that mentored the Government of India initiative Biotechnology Consortium of India Limited (BCIL) trainees during November 2012 to May 2013. WiB members also have shared posters and presentations during various conferences: a presentation on “Women in Biology Forum, Bioclues” at the BIFX′2013 virtual conference, May 3, 2013, www.bioinformatics.org; a poster on “Women in Biology: Bridging the Gender Gap to Innovation” at a national-level conference held at IKP Knowledge Park, Hyderabad, India, October 2012, www.ikpknowledgepark.com; a poster on “p53 and Systemic Lupus Erythematosus: A Putative Protein Interaction Analysis” at the 10th Latin American Association of Immunology (ALAI 2012), Lima, Peru, May 29–June 2, 2012, http://www.iuisonline.org/iuis/index.php/events/2-congress/3-10th-congress-of-alai.html; and a poster presentation on “p53 and the Cardiovascular System” at the BIFX′2012 virtual conference, March 1–2, 2012, www.bioinformatics.org.

WiB is grateful for the numerous women who have volunteered to organize and anchor the annual awards ceremony and participated in the essay writing competitions sponsored by Bioclues. During 2012–2013, there has been a significant improvement in the progress of WiB members at personal and scientific levels, be it project development, mentoring, or supporting open access. WiB believes its efforts have helped its members thrive, but they aim to see more women entrepreneurs and successful scientists at higher echelons. WiB also believes the latter can only be possible through greater financial support.

The growth and advancement of science can only be realized fully through the contributions of both men and women. WiB believes every community and every country should have similar groups at the local level to function effectively. WiB welcomes researchers from around the world to be a part of the team and contribute to this meaningful work.

